# Computational Insights into the Sequence-Activity Relationships of the NGF(1–14) Peptide by Molecular Dynamics Simulations

**DOI:** 10.3390/cells11182808

**Published:** 2022-09-08

**Authors:** Serena Vittorio, Candida Manelfi, Silvia Gervasoni, Andrea R. Beccari, Alessandro Pedretti, Giulio Vistoli, Carmine Talarico

**Affiliations:** 1 Dipartimento di Scienze Farmaceutiche, Università degli Studi di Milano, Via Mangiagalli, 25, I-20133 Milano, Italy; 2 Dompé Farmaceutici SpA, EXSCALATE, Via Tommaso De Amicis, 95, I-80131 Napoli, Italy; 3 Department of Physics, University of Cagliari, I-09042 Monserrato, Italy

**Keywords:** Nerve Growth Factor, TrkA, molecular dynamics, MM-GBSA, per-residue energy decomposition

## Abstract

The Nerve Growth Factor (NGF) belongs to the neurothrophins protein family involved in the survival of neurons in the nervous system. The interaction of NGF with its high-affinity receptor TrkA mediates different cellular pathways related to Alzheimer’s disease, pain, ocular dysfunction, and cancer. Therefore, targeting NGF-TrkA interaction represents a valuable strategy for the development of new therapeutic agents. In recent years, experimental studies have revealed that peptides belonging to the N-terminal domain of NGF are able to partly mimic the biological activity of the whole protein paving the way towards the development of small peptides that can selectively target specific signaling pathways. Hence, understanding the molecular basis of the interaction between the N-terminal segment of NGF and TrkA is fundamental for the rational design of new peptides mimicking the NGF N-terminal domain. In this study, molecular dynamics simulation, binding free energy calculations and per-residue energy decomposition analysis were combined in order to explore the molecular recognition pattern between the experimentally active NGF(1–14) peptide and TrkA. The results highlighted the importance of His4, Arg9 and Glu11 as crucial residues for the stabilization of NGF(1–14)-TrkA interaction, thus suggesting useful insights for the structure-based design of new therapeutic peptides able to modulate NGF-TrkA interaction.

## 1. Introduction

The Nerve Growth Factor (NGF) was first discovered in 1952 by Rita Levi Montalcini as a survival factor for sensory and sympathetic nerve growth during embryogenesis [1].

NGF elicits its physiological functions by interacting with two types of receptors: the low affinity p75 neurotrophin receptor (NTR) and the tropomyosin-related kinase A (TrkA). In particular, the interaction with TrkA induces receptor dimerization and autophosphorylation of different tyrosine residues, thus activating downstream pathways related to the expression of genes implicated in neuronal survival and differentiation [2,3].

Several studies pointed out that NGF promotes the survival of cholinergic neurons in Alzheimer’s disease (AD) [4], although its use in the therapy of AD is limited by its poor metabolic stability and low blood-brain barrier permeability [5]. Moreover, NGF exerts a protective and healing effect on the corneal tissue that led to the approval of recombinant human NGF (Oxervate, Dompé Farmaceutici SpA, Milan, Italy) for the topical treatment of neurotrophic keratopathy, a rare corneal disorder induced by trigeminal nerve impairments which causes epithelial damage and corneal ulcerations [6]. In addition, the activation of NGF-TrkA pathway is also implicated in the transmission of pain signals [7] as well as in tumorigenesis and metastasis formation in different types of cancer [8].

Due to its involvement in several biological processes, NGF-TrkA interaction represents an attractive target for pharmacological intervention in the therapy of AD, ocular dysfunctions, pain and cancer.

**Figure 1:**
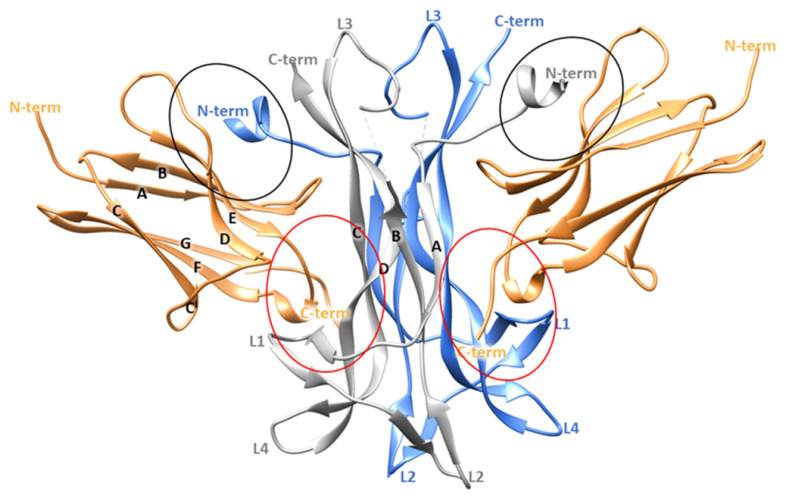
Crystallographic structure of TrkA-NGF complex (PDB ID 1WWW). The two NGF monomers are depicted as grey and blue ribbons while the domain 5 of TrkA is represented as sand-colored ribbons. The specific and the conserved patches are highlighted by red and black circles, respectively. The image was generated by using Chimera software V1.16 (Resource for Biocomputing, Visualization, and Informatics, University of California, San Francisco, CA, USA) [12].

From a structural point of view, NGF consists of two pairs of twisted antiparallel β -strands with three hairpin loops (L1, L2, L4) at one end, and a reverse turn (L3) and a cysteine knot motif at the other end. In the biologically active form, NGF is composed by two monomers assembled in parallel and linked by non-covalent interactions (Figure 1) [9,10]. Its binding partner TrkA presents three different domains: (i) the intracellular region containing the tyrosine kinase domain, (ii) the transmembrane domain and (iii) the extracellular domain. The last region comprises five subdomains: (i) a leucine-rich region (LRR, domain 2), (ii) two cysteine rich clusters (domains 1 and 3) and two immunoglobulin (Ig)-like domains (domains 4 and 5) [11]. NGF interacts with the domain 5 (d5) of TrkA which consists of a β-sandwich composed by two four stranded sheets: one formed by strands A, B, E, and D and the other by strands C′, C, F and G (Figure 1) [10].

Crystallographic studies provided important insights about the TrkA-NGF interaction, highlighting two ligand-receptor binding interfaces denoted as conserved patch and specific patch (Figure 1). The former comprises residues conserved among all the neurotrophins and the Trk receptors, while the latter involves non conserved residues located at the N-terminus of NGF (residues 2–13) and on the ABED sheet of TrkA-d5. Therefore, the N-terminal domain of NGF defines the specificity for the binding to TrkA [10].

Interestingly, TrkA-d5 in the unbound form displayed a similar conformation to that found in the complex with NGF, revealing that the interaction with the protein partner does not imply structural rearrangements. Conversely, significant conformational changes caused by receptor binding were observed in the N-terminus portion of NGF. Specifically, this region was disordered in the X-ray structures of NGF in its unbound state while it is well defined in the complex with TrkA, in which the residues 6–9 form a short helix [10,11]. Experimental and computational data revealed that the NGF N-terminal region plays a pivotal role in the binding and activation of TrkA [13,14,15,16]. Within this context, recent studies proved that a small peptide covering the 1–14 sequence of the human NGF and its acetylated and dimeric derivatives were able to activate TrkA, largely reproducing the effects induced by the whole protein [17,18,19]. Further experiments were performed by using two peptides characterized by the reverse, NGF(14-1), and the scrambled, s-NGF(1–14), amino acid sequence of NGF(1–14); both peptides were unable to mimic the biological activity of the native protein confirming the importance of the primary sequence in the activation of NGF-mediated pathways [19,20].

Taken together these results suggested that small peptides mimicking the NGF N-terminus portion could exert an NGF-like activity, thus allowing the improvement of the pharmacokinetic profile as well as the selective activation of targeted signaling pathways [21].

Basing on the above reported findings, our in silico study aims to analyze in depth the molecular recognition pattern between the N-terminal segment of NGF and TrkA, providing sequence-activity relationships useful for the rational design of peptide analogs. For this purpose, the dynamic behavior of the N-terminal region of NGF was explored by using molecular dynamics (MD) simulations [22,23]. In particular, three independent 200 ns MD runs were initially performed on the full-length protein in complex with TrkA and in its unbound state. The obtained outcomes corroborated the data reported in the literature [13,14] emphasizing the important role elicited by the NGF N-terminus in the binding to its receptor. Similarly, the experimentally active N-terminal peptide, NGF(1–14), was generated starting from the X-ray structure of NGF-TrkA complex and simulated in its unbound and bound forms, assuming that its binding mode is similar to that found in the full-length protein. The binding free energy of the simulated complexes was computed by MM-GBSA method and per-residue decomposition analysis was performed in order to identify the residues that mostly contribute to the stability of the studied systems. Basing on these results, four NGF(1–14) peptide mutants were simulated in order to further analyze how the mutations can affect its binding capability thus providing useful hints for structural modifications.

## 2. Materials and Methods

### 2.1. Protein Preparation and Molecular Dynamics Simulation

The crystallographic structure of NGF in complex with TrkA was retrieved from the wwwPDB (PDB ID 1WWW) [10] and used to provide initial coordinates for the generation of complex-S, complex-C, NGF(1–14)-TrkA complex, NGF and NGF(1–14). Missing residues were added to the protein by using VegaZZ software v 3.2.3.8 (Drug Design Laboratory, University of Milan, Milan, Italy) [24]. H++ webserver was used to add hydrogens and define both the tautomeric state of the histidine and the arrangement of asparagine and glutamine residues [25]. Concerning the systems involving the mutated peptides, the mutations were performed manually by using VegaZZ suite starting from the 3D coordinates of NGF(1–14)-TrkA complex. All the MD simulations were performed by using Amber v18 package (University of California, San Francisco, CA, USA) applying the following protocol [26]. The ff14SB forcefield [27] was used to parametrize the studied systems that were solvated in a box of TIP3P water molecules, whose size is reported in Table S1, employing a distance value of 10 Å and neutralized adding an appropriate number of Na+ and Cl- ions in order to reproduce a physiological salt concentration of 0.15 M. The generated systems underwent three steps of energy minimization involving first the hydrogen atoms, then the water molecules and finally the side chains. After the minimization, a heating phase of 20 ps was performed in which the temperature was increased from 0 to 300 K by using the Langevin thermostat [28] and applying positional restraints (5 Kcal/mol) on the Cα atoms. The equilibration was conducted in NPT ensemble for 50 ps maintaining the restraints on the Cα atoms and then for 70 ps by gradually reducing the weight of the restraints. The pressure was kept around 1 atm by employing a Berendsen barostat [29]. Finally, 200 ns of production run were performed at constant pressure without any restraint. All the bonds involving hydrogen atoms were restrained by SHAKE algorithm [30] using a timestep of 2 fs. Electrostatic interactions were computed by particle-mesh Ewald (PME) method [31] and periodic boundary conditions were applied. All the simulations were run in triplicate starting from the same initial coordinates but with different random seeded velocities. The coordinates were stored every 20 ps resulting in 10,000 frames. The cpptraj module of Amber v18 [32] was employed for the analysis of the resulting trajectories.

### 2.2. Binding Free Energy Calculations and Per-Residue Energy Decomposition Analysis

The binding free energy of the simulated complexes was computed by using the molecular mechanics/generalized born solvent accessibility (MM/GBSA) method implemented in Amber18 as described elsewhere [33]. The calculation was carried out for all the resulting frames (10,000) of each independent MD run performed for each system, and the obtained energies were averaged.

## 3. Results and Discussion

### 3.1. MD Simulation of NGF-TrkA Complex

**Figure 2:**
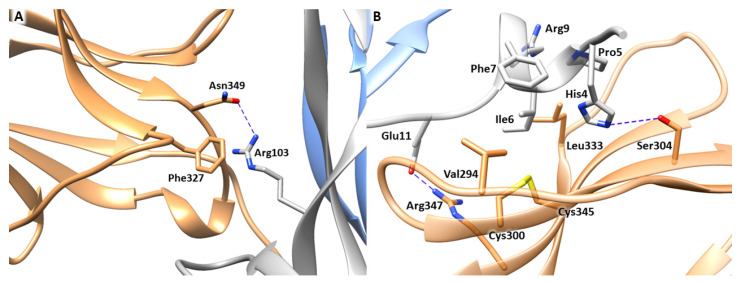
Binding interfaces of NGF-TrkA complex. (**A**) Close view of the conserved patch. (**B**) Close view of the specific patch. The key residues of the two binding interfaces are represented as grey (NGF) and sand-colored (TrkA) sticks. H-bond interactions are depicted as blue dashed lines. The image was created by means of Chimera software.

The first step of our computational study involved the analysis of the experimentally resolved structure of NGF-TrkA complex (PDB ID 1WWW), in which the NGF homodimer contacts two copies of TrkA-d5 domain forming a symmetrical assembly [10]. As reported above, two main binding sites, known as conserved and specific patches, can be identified along the protein-protein interface (Figure 1). The conserved patch includes the C-terminal loop of TrkA-d5 and the β-hairpin loop L1 and the central β-sheet of NGF. In this region, the side chain of Arg103 of NGF stacks against the aromatic ring of Phe327 of TrkA and engages a H-bond with Asn349 (Figure 2A). Instead, the specific patch comprises the ABED sheet of TrkA and the NGF N-terminal segment which contains the residue His4 that is known to have a crucial role in the receptor binding (Figure 2B) [15]. Mutagenesis assays revealed that the mutations of His4 to alanine and aspartate significantly reduced TrkA binding; moreover, experimental studies highlighted that the deletion of the first nine residues of NGF resulted in affinity loss of about 300 fold [13]. Notably, this effect is not related to conformational changes of NGF suggesting the functional role of the N-terminal residues in receptor binding [14]. At a molecular level, the N-terminus of NGF interacts with TrkA establishing two H-bonds between (i) His4 of NGF and Ser304 of TrkA and (ii) Glu11 of NGF and Arg347 of TrkA (Figure 2B). Furthermore, Ile6 of NGF occupies a hydrophobic niche lined by Val294, Leu333 and the disulfide bridge formed by Cys300 and Cys345 of TrkA. Additional hydrophobic contacts are engaged by Pro5 and Phe7 of NGF.

**Figure 3:**
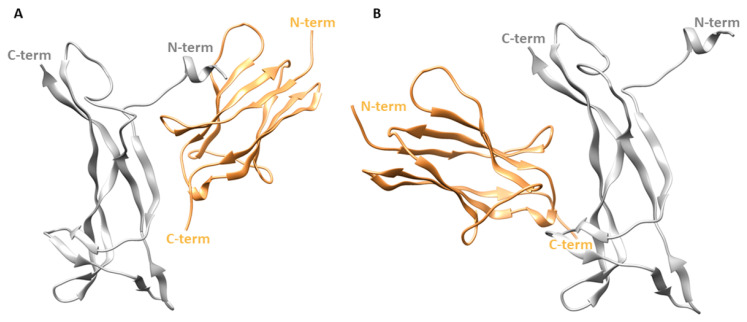
Structures of the two simulated complexes. (**A**) Complex-S comprising the specific patch as binding interface. (**B**) Complex-C including the conserved patch. NGF is represented as gray ribbons while TrkA is displayed as sand-colored ribbons. The image was created by using Chimera software.

In the X-ray structure, the two patches of one NGF molecule contact different TrkA-d5 monomers as displayed in Figure 1. In order to independently evaluate the contribution of each patch in the stabilization of NGF-TrkA interaction, two complexes were generated: the first one, named “complex-S”, included the specific patch as binding interface (Figure 3A), while the second one, termed “complex-C” contained the conserved patch (Figure 3B). Both complexes were subjected to three independent 200 ns MD runs performed by using the software Amber v18 [26]. Moreover, the same computational protocol was applied to NGF in its unbound state.

**Figure 4:**
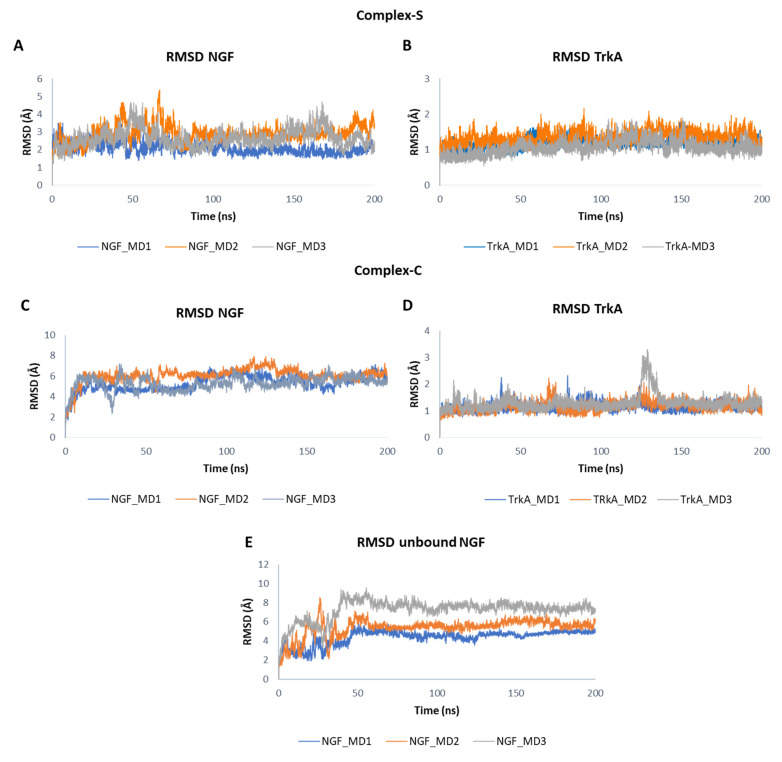
RMSD plots of NGF and TrkA backbones registered in the three MD runs performed on complex-S (**A**,**B**), complex-C (**C**,**D**) and the unbound NGF (**E**).

In Figure 4 the root mean square deviation (RMSD) plots related to the backbones of TrkA and NGF in the three simulated systems are displayed. The results showed that TrkA is characterized by a lower structural mobility if compared to NGF. Furthermore, its dynamic behavior is not affected by the binding to NGF considering that similar RMSD profiles were obtained in the two complexes although they are characterized by different interaction patterns. In contrast, the binding to TrkA exerts a stabilizing effect on NGF especially in the “complex-S” in which lower RMSD values were monitored in respect to those obtained for the other two simulated systems. Overall, in complex-S, NGF showed a stable profile in all the three trajectories with mean RMSD values of 2.10 Å, 2.91 Å and 2.65 Å in the MD1, MD2 and MD3, respectively (Figure 4A). A greater mobility was observed for NGF in complex-C in which the computed averaged RMSD values were 5.31 Å, 6.07 Å and 5.27 Å.

When simulated alone, the RMSD profile of NGF showed a similar trend in the three trajectories. In detail, after initial fluctuations, the RMSD converged at about 50 ns and remained stable for the rest of the simulation (Figure 4E). However, the system reached stability at different RMSD values, assuming mean values of 4.38 Å in MD1, 5.38 Å in MD2 and 7.23 Å in MD3.

**Figure 5:**
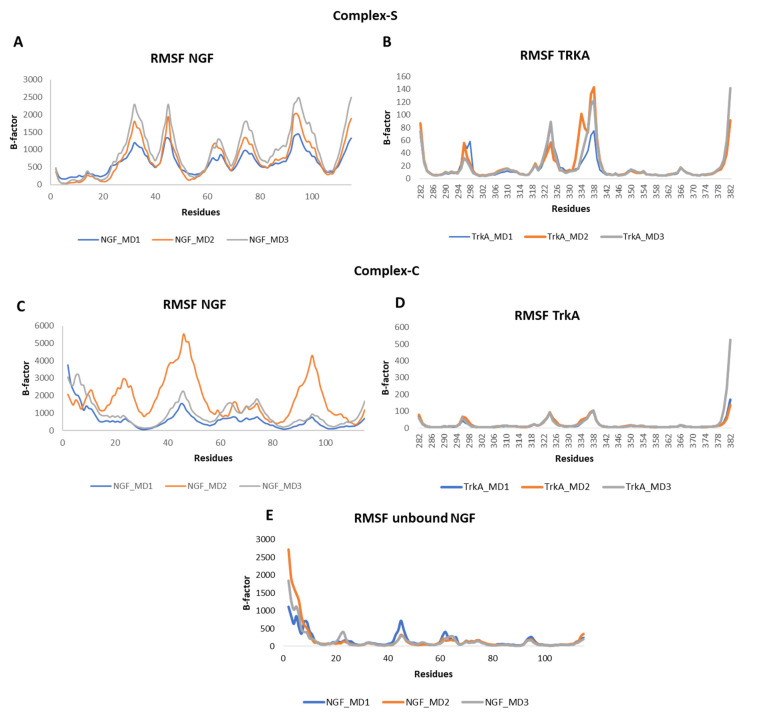
RMSF profiles of NGF and TrkA backbones registered in the three MD runs performed on complex-S (**A**,**B**), complex-C (**C**,**D**) and the unbound NGF (**E**).

The root mean square fluctuation (RMSF) of the backbone atoms of the two proteins was calculated for each simulation (Figure 5). Concerning NGF, in all the three systems we could observe some flexibility around residues 45 and 95 which belong to L2 and L4, respectively. Other flexible regions were detected in complex-S and in complex-C around residues 65 and 75, both located on L3 of NGF. As expected, in complex-S (Figure 5A) the N-terminal region showed a lower flexibility if compared to both complex-C and the unbound NGF (Figure 5C,E) due to its interaction with TrkA. Moreover, in complex-S the region around residue 30 showed a high mobility, different from the other two simulated systems. Instead, no significant differences were observed in the RMSF profile of TrkA in the two complexes, further emphasizing that the binding of NGF does not induce any significant structural rearrangement in TrkA. Notably, the more flexible regions were detected around residues 296, 324 and 337, located on three different loops (Figure 5B,D).

Secondary structural analysis of NGF was performed for the three simulated systems by using DSSP algorithm. The outcomes obtained for one MD trajectory for each system are reported in Supplementary Material (Figures S1–S3). As expected, the main difference relies on the conformations adopted by the N-terminal domain of NGF which assumes mostly an alpha helix conformation in complex-S (Figure S1), while forming turn structures in complex-C (Figure S2). Interestingly, in solution, the N-terminus of NGF is characterized by the propensity to form turns and 3–10 helix (Figure S3).

To estimate the binding free energy of the two complexes, we performed MM-GBSA calculations on each MD output and the mean values over the three MD runs were computed (Table 1). The outcomes showed that complex-S is more stable than complex-C, with a mean ΔG value (−55.44 Kcal/mol) more than two-fold lower than that of complex-C (−20.81 Kcal/mol). In light of this, per-residue free energy decomposition analysis was carried out for complex-S to evaluate the contribution of each residue to the binding free energy. By averaging the results obtained from the three MD simulations, we observed that the most favorable energy contributions derived mainly from the residues located at the N-terminal region of NGF and, in particular, from His4, Ile6, and Arg9 whose contribution to the ΔG is lower than −3.0 Kcal/mol (Figure S4). Other significant contributions derived from Trp21 and Arg59 belong to the central β-sheet of NGF.

To clarify the role of each of these residues at atomic level, the distances between the interacting amino acids were monitored in each MD run and the results were averaged (Table S2). The obtained outcomes revealed that His4 elicited persistent H-bonds with the backbone of Phe303 and Gly344, and, to a lesser extent, with the side chains of Ser304 and His343. Moreover, it also engages beneficial hydrophobic contacts with His291 and Pro302. Instead, Leu6 is involved in stable hydrophobic interactions with the disulfide bridge formed by Cys300 and Cys345, while Pro5 and Phe7 interact with Leu333 and Val294, respectively. Arg9 established a salt bridge with Glu334 which showed itself to be more stable in the MD runs 1 and 3, respect to the MD2. A similar behavior was also detected for the salt bridge between Arg59 and Glu295. Instead, Trp21 created a network of H-bonds involving His253 and Asp380. Finally, other significant interactions were mediated by Glu11 of NGF which engaged a H-bond with His297 and a salt bridge with Arg347.

Overall, our computational analysis performed on the X-ray structure of NGF-TrkA complex is in agreement with the experimental data reported to date in the literature and confirms that the N-terminal segment of NGF affords the most relevant contribution for the interaction with TrkA.

### 3.2. MD Simulation of NGF(1–14)-TrkA Complex

As mentioned above, experimental studies revealed the ability of a NGF peptide encompassing the 1–14 sequence to partly mimic the biological activity of the native protein, paving the way to new opportunities for the rational design of small therapeutic peptides able to modulate NGF-TrkA pathways [17].

To get more insights about the molecular recognition pattern between NGF(1–14) and TrkA, MD simulations were performed on this complex by applying the same protocol used for the X-ray structure and by assuming that the peptide adopts a similar binding mode to that assumed by this protein portion in the complex involving the full-length protein. For easy comparison, MD studies were also carried out on the peptide NGF(1–14) in its unbound state.

**Figure 6:**
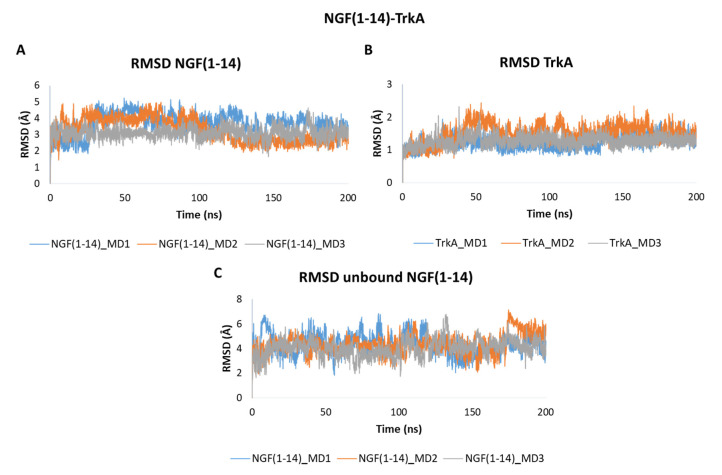
RMSD plots of the backbones of (**A**) NGF(1–14) and (**B**) TrkA registered during the three MD runs performed on NGF(1–14)-TrkA complex. (**C**) RMSD of NGF(1–14) backbone simulated in the unbound form.

The obtained outcomes pointed out that NGF(1–14) forms a stable adduct with TrkA as showed by the RMSD plots in Figure 6. In detail, NGF(1–14) exhibits a stable behavior in all the three trajectories registering mean RMSD values of 3.66 Å, 3.28 Å and 3.10 Å in MD1, MD2 and MD3, respectively (Figure 6A). As expected, TrkA displayed RMSD profiles (Figure 6B) similar to those observed for complex-S and complex-C, thus confirming that its dynamic behavior is not strongly influenced by its interaction partner. When simulated alone, the peptide showed a greater flexibility with average RMSD values of 4.33 Å in both MD1 and MD2, and of 4.04 Å in MD3 (Figure 6C).

**Figure 7:**
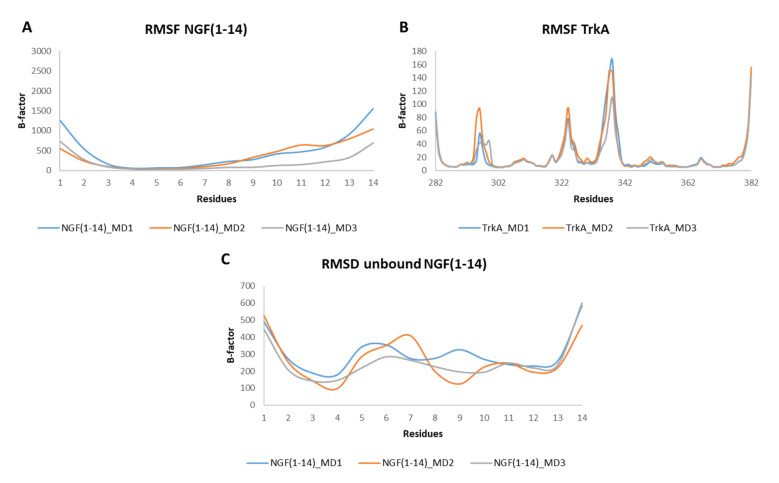
RMSF plots of the backbones of NGF(1–14) (**A**) and TrkA (**B**) registered during the three MD runs performed on NGF(1–14)-TrkA complex. (**C**) RMSF profiles of NGF(1–14) backbone.

The RMSF profiles of NGF(1–14) in complex with TrkA revealed that residues 4–6 are characterized by a low mobility because they are involved in stable interactions with the receptor. In contrast, a greater flexibility was detected for the segment 8–14 (Figure 7A). Regarding TrkA, the most flexible regions were those around residues 296, 324 and 337 (Figure 7B) as seen previously for complex-S and complex-C. Instead, concerning the unbound NGF(1–14), the most flexible regions were those around residues 6 and 11. Moreover, some flexibility was detected around (i) Phe7 in MD2 and (ii) Arg9 in MD1 (Figure 7C).

Binding free energy calculations were performed for NGF(1–14)-TrkA complex by using the MM-GBSA method yielding a mean value, computed over the three trajectories, equals to −40.53 Kcal/mol (Table 1). The obtained ΔG was higher than that calculated for complex-S but lower than that registered for complex-C. In Figure S5, the contribution of each energy term to the binding free energy is displayed for each complex. Notably, the electrostatic energy term is comparable in the two complexes involving the full-length NGF, while being higher in the adduct with the N-terminal peptide. The unfavorable increment of the electrostatic energy in the NGF(1–14)-TrkA complex is overcompensated by the reduction of the polar solvation energy that led to a favorable shift of the ΔG if compared to the complex-C. Instead, the lowest ΔG of complex-S is ascribable to the lower van der Waals energy with respect to the other simulated systems (Figure S5).

Per-residue energy decomposition was carried out on each MD run of the NGF(1–14)-TrkA complex and the results were averaged. The outcomes revealed that His4, Ile6 and Arg9 furnished the most significant contribution to the binding free energy (Figure S6) with mean ΔG values lower than −3 Kcal/mol, thus confirming the results gained for the full-length NGF in complex-S.

To better understand the role of these residues, the distances between the key residues at the protein-peptide interface were calculated during the three MD runs (Table S3). Overall, the results showed that NGF(1–14) is able to maintain the same interactions that this protein portion established in complex-S. The main differences concern the distances registered between (i) Arg9 and Glu334, (ii) Glu11 and Arg347 and (iii) Glu11 and His297, whose mean values increase by 2.57 Å, 5.61 Å and 4.57 Å, respectively. Therefore, differently from complex-S, Arg9 and Glu11 are not able to elicit stable interactions with TrkA and this explains the high flexibility detected for the segment 8–14 of NGF(1–14) during the MD trajectories.

Considering that peptides are high flexible entities, their propensity to adopt the bioactive conformation must be considered during drug design efforts. To this end, the secondary structural propensity for NGF(1–14) in both the bound and unbound states was monitored during the simulations. The outcomes revealed that when in complex with TrkA, the residues 5–7 of NGF(1–14) mainly adopted a 3–10 helix structure (Figure S7A). Instead, His8 and Arg9 initially assumed an alpha helix conformation that is converted, respectively, into turn and bend during the trajectory (Figure S7B). The other residues mostly adopted unstructured motifs, except for the segment 11–12 for which the propensity to form turns and 3–10 helix was observed during the simulation.

**Figure 8:**
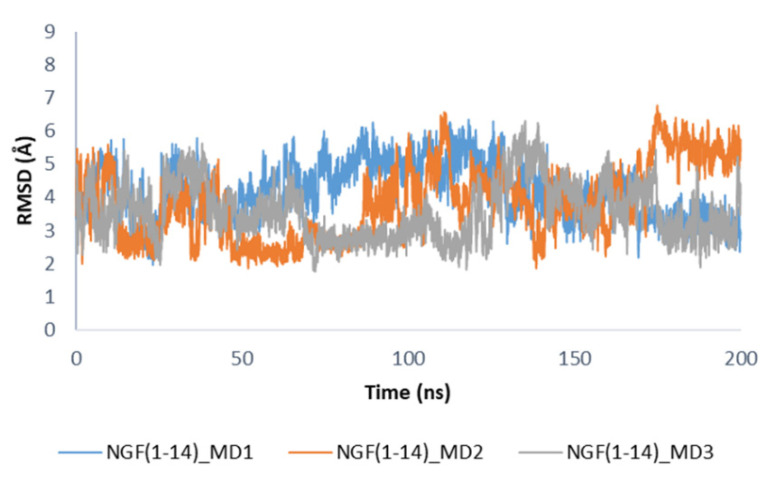
RMSD profiles of NGF(1–14) backbone in the unbound state calculated by using the conformation of the peptide in complex with TrkA as reference structure.

Concerning the secondary structure analysis of the unbound NGF(1–14) in solution, as expected some regions were more unstructured if compared to the bound form as displayed in Figure S8. In this case, residues 5–8 mainly assumed an alpha helix conformation, while the propensity of Glu11 and Phe12 to form a 3–10 helix as observed in the bound state is decreased. It is worthwhile to note that in solution the peptide is able to assume the structural motifs observed for these protein portions in the X-ray structure. The conformations registered during the MD simulations of NGF(1–14) in solution were compared to that assumed by the peptide in the complex with TrkA. We observed that, among the collected conformations, about 11% in MD1, 34% in MD2 and 41% in MD3, were similar (RMSD = 2–3 Å) to that observed in the complex with TrkA (Figure 8), thus underlining the capability of NGF(1–14) to assume conformations suitable for the binding to the target protein.

### 3.3. MD Simulations of TrkA in Complex with NGF(1–14) Mutants

The results of our computational study performed on NGF(1–14)-TrkA complex revealed that residues His4, Ile6 and Arg9 mediated the most stable interactions and provided the most favorable contributions to the binding free energy. Basing on these results, we further investigate the crucial role of these residues by performing MD simulations on TrkA in complex with NGF(1–14) mutants in which these amino acids were mutated to Ala. Furthermore, we also decided to explore the effect of the mutation on the other charged residue of NGF(1–14), namely Glu11.

**Figure 9:**
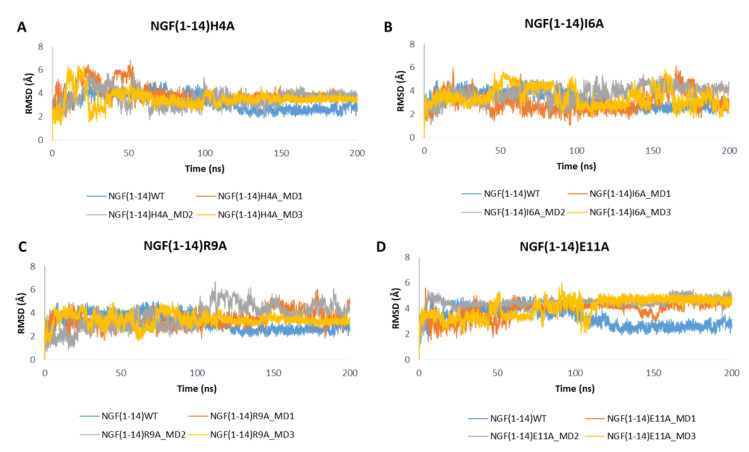
RMSD profiles of the backbone of NGF(1–14) mutants H4A (**A**), I6A (**B**), R9A (**C**) and E11A (**D**) obtained for each MD run.

Figure 9 depicts the RMSD plots related to the backbones of the mutated peptides. For easy comparison, the RMSD profile of the wild-type peptide obtained in one of the MD runs performed on NGF(1–14)-TrkA complex is also displayed. Overall, the outcomes show that the mutations H4A and R9A led to a slight increase of the RMSD values with respect to the native NGF(1–14) (Figure 9A,C), while a more appreciable increment could be observed for the E11A mutant (Figure 9D). Concerning the I6A mutant, aside from some fluctuations, the RMSD profiles of MD1 and MD3 are similar to that of the wild-type peptide; instead, in the MD2, the RMSD graph of NGF(1–14)I6A is superimposable to that of the native form in the first part of the trajectory, while in the second part the mutated peptide reached the stability at higher RMSD values, if compared to the WT form (Figure 9B).

**Figure 10:**
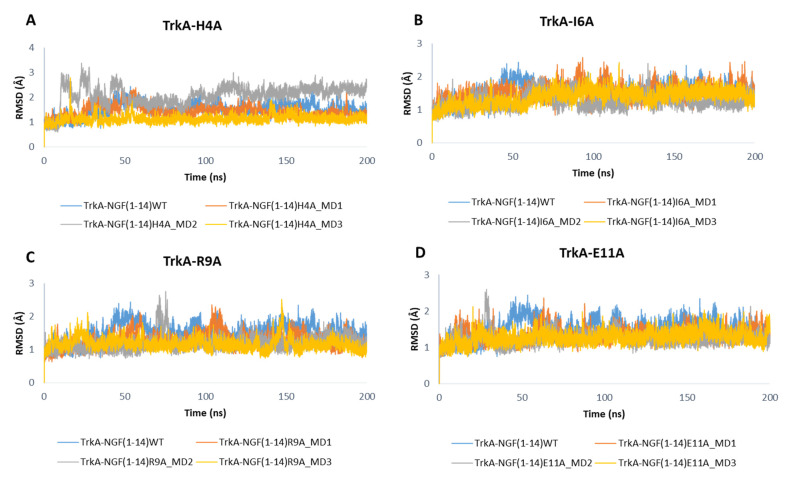
RMSD profiles of TrkA backbone in complex with NGF(1–14) mutants H4A (**A**), I6A (**B**), R9A (**C**) and E11A (**D**).

As displayed in Figure 10, the mutations did not affect the dynamic behavior of TrkA, whose RMSD profiles are similar to those obtained for the previous simulated systems. Only the mutations H4A might affect TrkA stability as observed in the MD2 of NGF(1–14)H4A-TrkA complex.

**Figure 11:**
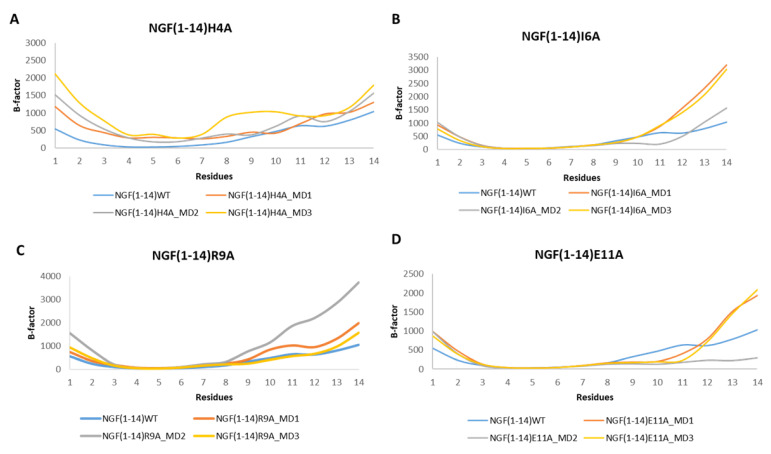
RMSF profiles of the NGF(1–14) mutants H4A (**A**), I6A (**B**), R9A (**C**) and E11A (**D**) obtained for each MD run.

The analysis of the RMSF plots revealed that the mutation H4A led to an increment of the flexibility for almost all the residues in respect to NGF(1–14) (Figure 11A). Regarding the other mutants, the RMSF plots corroborated with the outcomes gained for the WT peptide with the residues 8–14 characterized by a high flexibility.

The RMSF profiles registered for TrkA in complex with the NGF(1–14) mutants are similar to those observed for the previous simulated systems and are depicted in Figure S9.

The impact of the mutations on the binding free energy was investigated by performing MM-GBSA calculations on the MD outputs of the complexes involving the mutated peptides. In this case, the ΔG was computed for each MD run and the results were averaged. The difference in the binding free energy between the WT and the mutants was computed on the mean values as follows: ΔΔG = ΔG mutant − ΔG wild-type, where ΔΔG values >0 indicates a destabilizing effect of the mutation on the protein-peptide complex. The obtained results are summarized in Table 2. The different energy components calculated from each MD simulation were also averaged and reported in Figure S10. As expected, the mutation H4A had a negative impact on the complex stability with a ΔΔG value of 6.98 kcal/mol that is mainly due to the increment of the van der Waals and electrostatic energy terms in respect to the WT peptide (Figure S10). Similarly, the mutations R9A and E11A unfavorably affected the binding free energy increasing the ΔG values by 10.63 and 5.27 kcal/mol, respectively. In the first case, we observed an increment of the electrostatic and van der Waals energies that was not overcompensated by the reduction of the polar solvation energy. Instead, the mutation E11A induced a decrease of the electrostatic energy that was not enough to compensate the unfavorable increase of the polar solvation energy (Figure S10). In contrast, the mutation I6A did not exert a significant impact on the binding free energy with a ΔΔG value of 0.55 kcal/mol, a result understandable by considering the rather conservative character of the simulated mutation.

## 4. Conclusions

This study described a computational workflow aimed at understanding the recognition pattern between TrkA and the N-terminal peptide of NGF, NGF(1–14), which proved to be able to largely mimic the effect of the full-length protein, paving the way towards new opportunity for the development of therapeutic peptides with a NGF-like activity and an improved pharmacokinetic profile. For this purpose, MD simulations and binding free energy calculations were applied to NGF(1–14)-TrkA complex, allowing the role of each residue in the complex stabilization to be elucidated. The obtained results highlighted that the residues 4–6 mediate the most stable interactions and provide the most important contribution to the binding free energy. Furthermore, the simulation of the unbounded NGF(1–14) in the water environment was also performed revealing that, despite its intrinsic flexibility, the peptide is able to assume during the trajectories conformations that are similar to that of the bound form. In this context, the present study can be also exploited to propose a computational workflow able to predict the reliability of new analogs of NGF(1–14). In such a workflow, the first step involves MD simulation on the designed peptide alone to analyze its propensity to adopt during the trajectory the binding conformation of NGF(1–14) used as template. Once that the capability of the peptide to adopt the bioactive conformation has been assessed, its ability to interact with the target can be evaluated by performing MD simulation on the complex using the here reported computational procedures. To this aim, the peptide could be forced to assume a binding pose analogous to that of NGF(1–14) to simplify the docking procedure.

Basing on the results gained from the MD simulations performed on NGF(1–14)-TrkA adduct, four peptide mutants H4A, I6A, R9A and E11A, were simulated in complex with TrkA to investigate the impact of the mutations on the complex stability, thus allowing us to investigate the sequence-activity relationship of NGF(1–14). The outcomes revealed that all the mutations, except I6A, caused an unfavorable shift of the binding free energy, providing new useful structural insights that can be applied to guide the structure-based design of NGF(1–14) mimetics for the development of new therapeutic agents.

## Figures and Tables

**Table 1:** ΔG values computed from each MD output of complex-S, complex-C and NGF(1–14)-TrkA complex, and mean values calculated by averaging the energies obtained from the three runs.

System	ΔG (Kcal/mol)			Mean
	MD1	MD2	MD3	
Complex-S	−53.07 ± 9.01	−55.56 ± 8.99	−54.68 ± 10.41	−54.44 ± 9.47
Complex-C	−20.99 ± 5.95	−22.32 ± 9.99	−19.11 ± 7.55	−20.81 ± 7.83
NGF(1–14)-TrkA	−40.49 ± 11.35	−38.28 ± 8.30	−42.82 ± 8.58	−40.53 ± 9.41

**Table 2:** ΔG values calculated from each MD run performed on TrkA in complex with the mutated NGF(1–14) and impact of the mutation on the binding free energy.

Systems	ΔG (kcal/mol)				ΔΔG
	MD1	MD2	MD3	Mean	
TrkA-NGF(1–14)WT	−40.49 ± 11.35	−38.28 ± 8.30	−42.82 ± 8.58	−40.53 ± 9.41	-
TrkA-NGF(1–14)H4A	−34.74 ± 11.82	−34.70 ± 11.01	−31.22 ± 7.05	−33.55 ± 9.96	6.98 ± 4.40
TrkA-NGF(1–14)I6A	−40.34 ± 8.68	−40.95 ± 7.78	−38.66 ± 9.98	−39.98 ± 8.81	0.55 ± 4.27
TrkA-NGF(1–14)R9A	−28.99 ± 5.72	−31.05 ± 6.47	−29.67 ± 5.20	−29.90 ± 5.80	10.63 ± 3.90
TrkA-NGF(1–14)E11A	−37.27 ± 6.66	−33.10 ± 6.80	−35.41 ± 7.61	−35.26 ± 7.02	5.27 ± 4.05

## Data Availability

All the relevant data are included in the manuscript.

## Supplementary Materials

The following supporting information can be downloaded at: https://www.mdpi.com/article/10.3390/cells11182808/s1, Table S1: Water box sizes of each simulated system; Figure S1: Secondary structural analysis for NGF in complex-S; Figure S2: Secondary structural analysis for NGF in complex-C; Figure S3: Secondary structural analysis for NGF simulated in the unbound state; Figure S4: Per-residue energy decomposition analysis performed by averaging the results gained from each MD simulation executed on complex-S; Table S2: Distances profiles of the key interactions detected during the three MD runs performed on Complex-S; Figure S5: Contribution of each energy term to the binding free energy of complex-S, complex-C and TrkA-NGF(1–14) obtained by averaging the results gained from each MD runs performed on the three complexes; Figure S6: Per-residue energy decomposition results performed on TrkA-NGF(1–14) complex by averaging the outcomes obtained from each MD run; Table S3: Distances profiles of the key interactions detected during the three MD runs performed on NGF(1–14)-TrkA complex; Figure S7: Secondary structural analysis for NGF(1–14) in complex with TrkA; Figure S8: Secondary structural analysis for NGF(1–14) in aqueous solution; Figure S9: RMSF profiles of TrkA in complex with the mutated peptides; Figure S10: Contribution of each energy term to the binding free energy of the complexes involving the mutated peptides.
