# Supplementary material for: Computational Insights into the Sequence-Activity Relationships of the NGF(1–14) Peptide by Molecular Dynamics Simulations
Source: Cells. 2022 Sep 8;11(18):2808. doi: 10.3390/cells11182808 (PMC9497175; doi:10.3390/cells11182808)

Supplementary Material

Computational Insights into the Sequence-Activity Relationships of the NGF(1-14) Peptide by Molecular Dynamics Simulations

Serena Vittorio [1], Candida Manelfi [2], Silvia Gervasoni [1,3], Andrea R. Beccari [2], Alessandro Pedretti [1], Giulio Vistoli [1] and Carmine Talarico [2,*]

[1] Dipartimento di Scienze Farmaceutiche, Università degli Studi di Milano, Via Mangiagalli, 25, I-20133 Milano, Italy

[2] Dompé Farmaceutici SpA, EXSCALATE, Via Tommaso De Amicis, 95, I-80131 Napoli, Italy

[3] Department of Physics, University of Cagliari, I-09042 Monserrato, Italy

*Correspondence: carmine.talarico@dompe.com

Content:

Table S1. Water box sizes of each simulated system 2

Figure S1. Secondary structural analysis for NGF in complex-S. 3

Figure S2. Secondary structural analysis for NGF in complex-C. 4

Figure S3. Secondary structural analysis for NGF simulated in the unbound state 5

Figure S4. Per-residue energy decomposition analysis performed by averaging the results gained from each MD simulation executed on complex-S. 6

Table S2. Distances profiles of the key interactions detected during the three MD runs performed on complex-S.. 7

Figure S5. Contribution of each energy term to the binding free energy of complex-S, complex-C and TrkA-NGF(1-14) obtained by averaging the results gained from each MD runs performed on the three complexes. 8

Figure S6. Per-residue energy decomposition results performed on TrkA-NGF(1-14) complex by averaging the outcomes obtained from each MD run. 9

Table S3. Distances profiles of the key interactions detected during the three MD runs performed on NGF(1-14)-TrkA complex.. 10

Figure S7. Secondary structural analysis for NGF(1-14) in complex with TrkA. 11

Figure S8. Secondary structural analysis for NGF(1-14) in aqueous solution. 12

Figure S9. RMSF profiles of TrkA in complex with the mutated peptides. 13

Figure S10. Contribution of each energy term to the binding free energy of the complexes involving the mutated peptides.. 14

Table S1. Water box sizes of each simulated system.

System	Water box size (Å)
Complex-S	96.13x63.13x93.52
Complex-C	87.77x59.86x99.88
Unbound NGF	87.30x56.32x73.27
Unbound NGF(1-14)	72.54x58.08x72.23
TrkA-NGF(1-14)	82.22x69.33x82.18
TrkA-NGF(1-14) mutants	82.22x 69.33x 82.18

Figure S1. Secondary structural analysis for NGF in complex-S. A) Average structural propensity over all frames for each residue. B) Time evolution of the secondary structure for each residue.

A

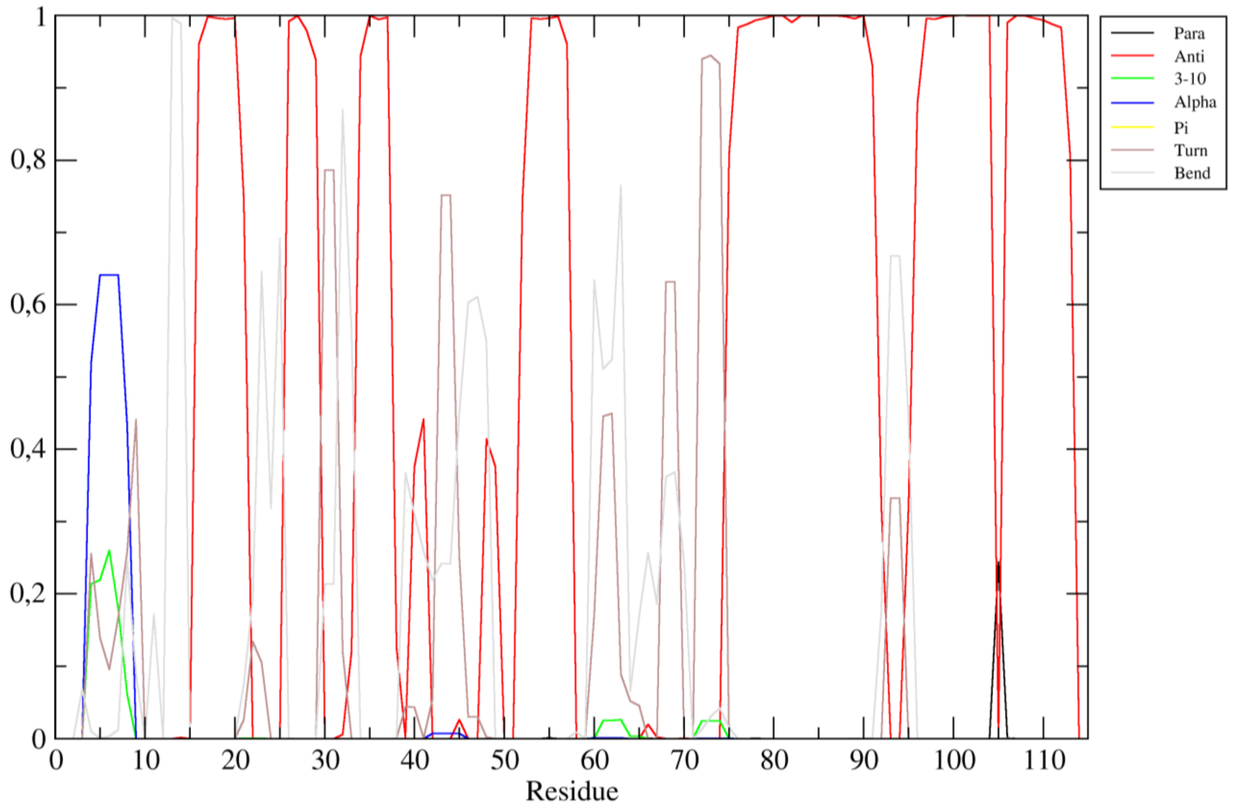

B

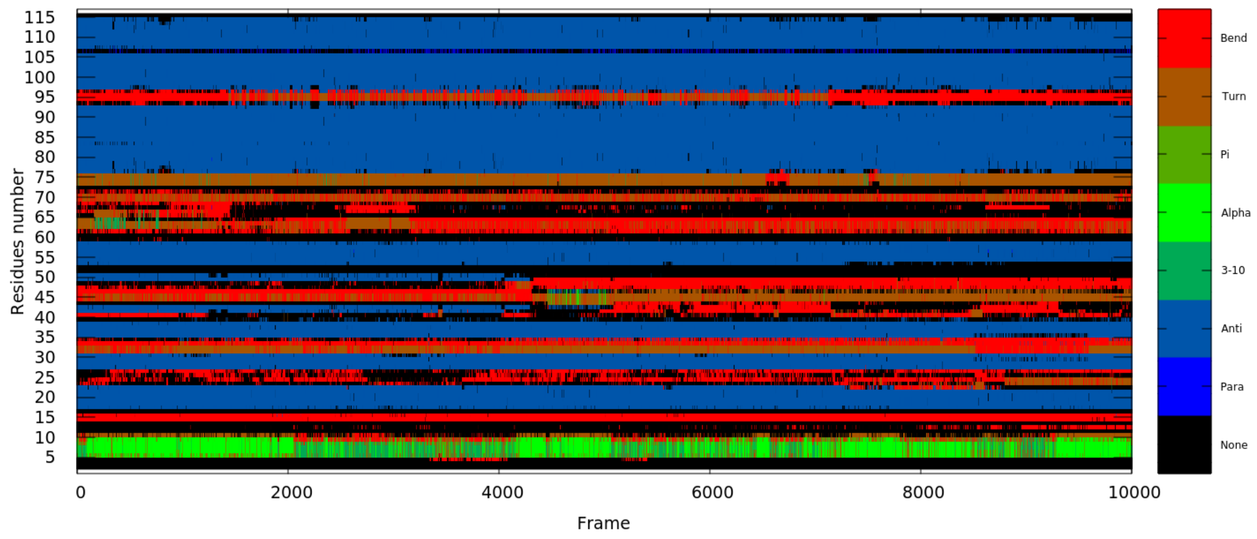

Figure S2. Secondary structural analysis for NGF in complex-C. A) Average structural propensity over all frames for each residue. B) Time evolution of the secondary structure for each residue.

A

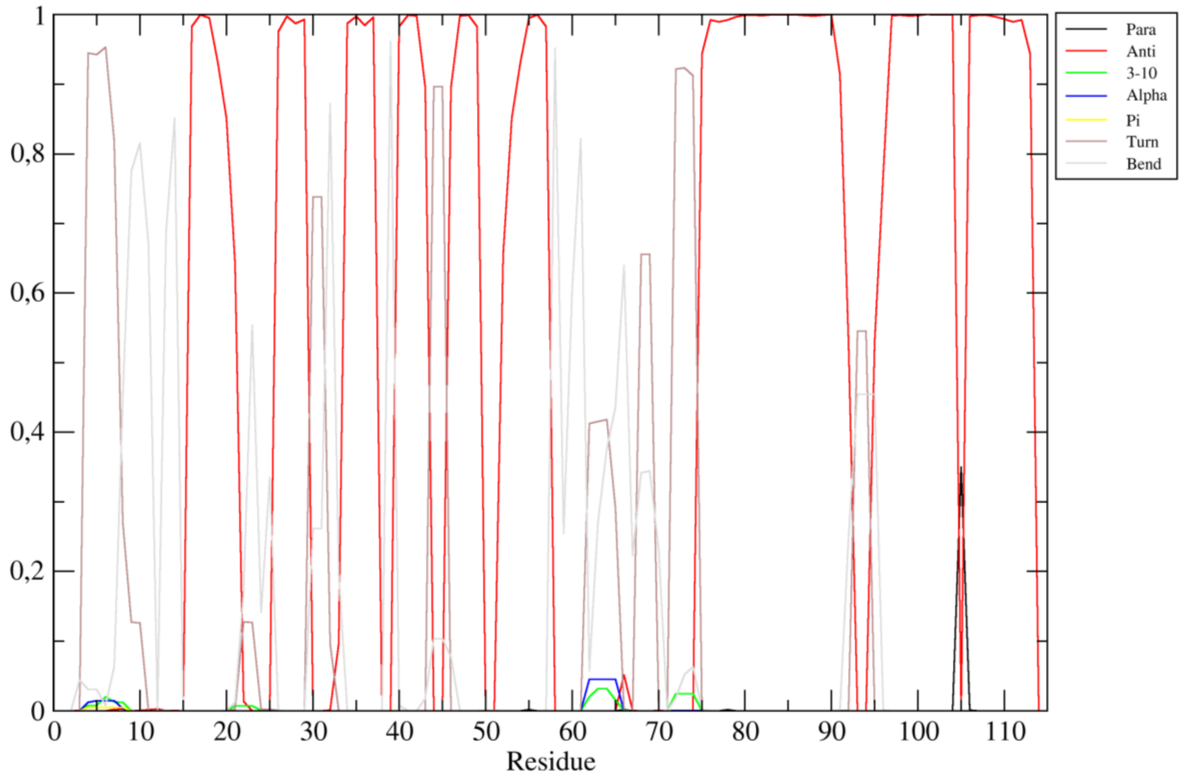

B

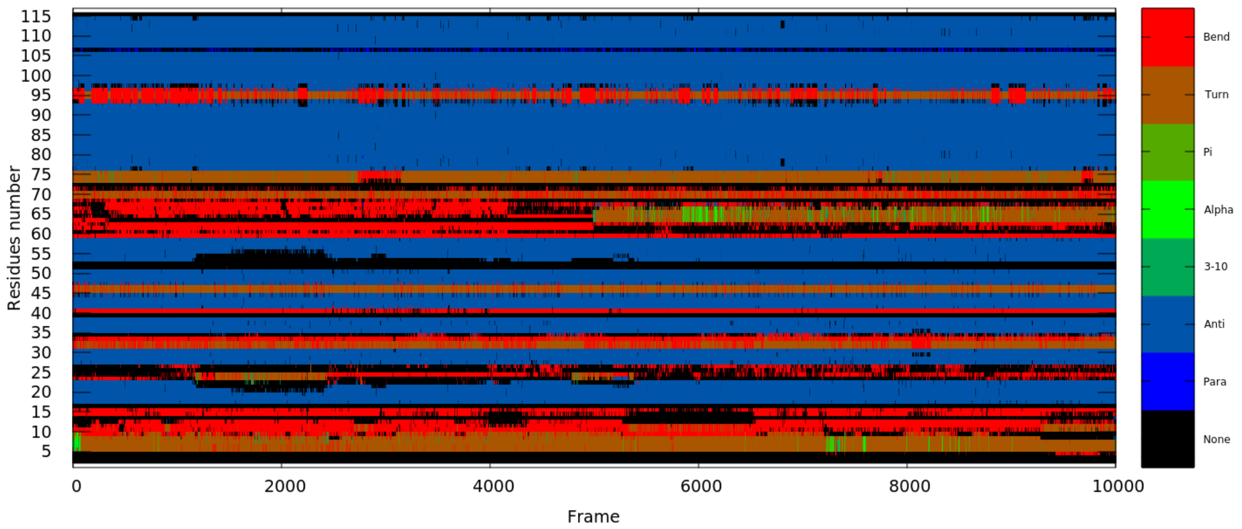

Figure S3. Secondary structural analysis for NGF simulated in the unbound state. A) Average structural propensity over all frames for each residue. B) Time evolution of the secondary structure for each residue.

A

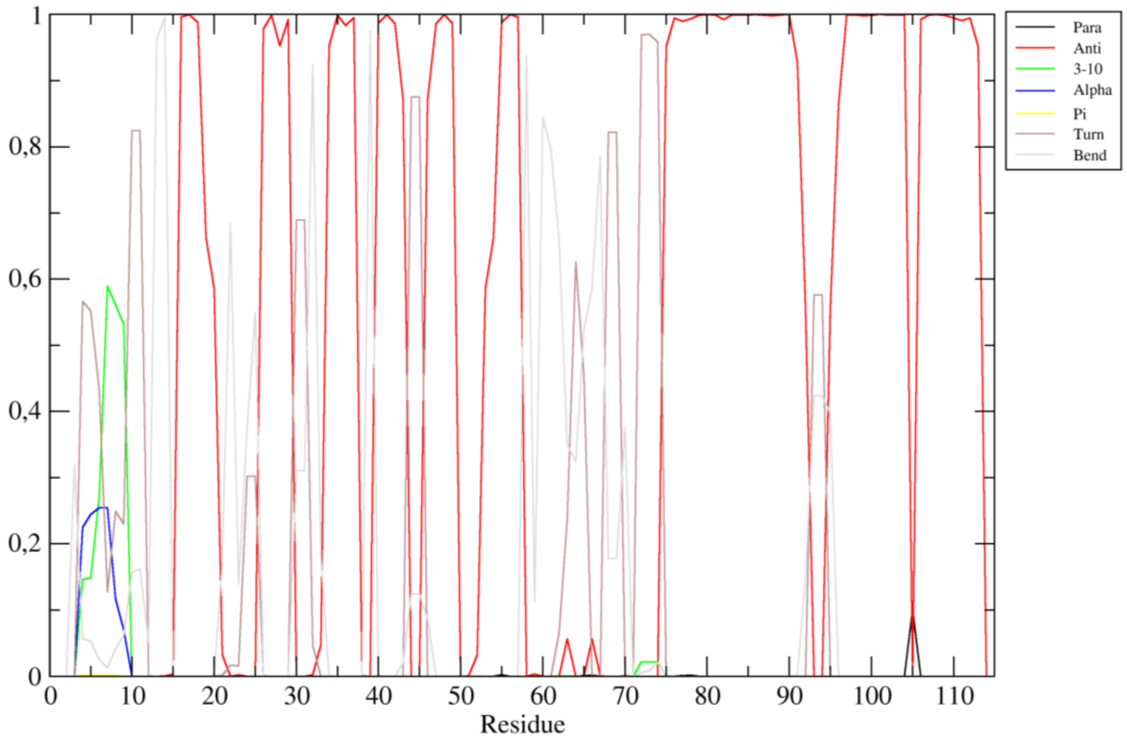

B

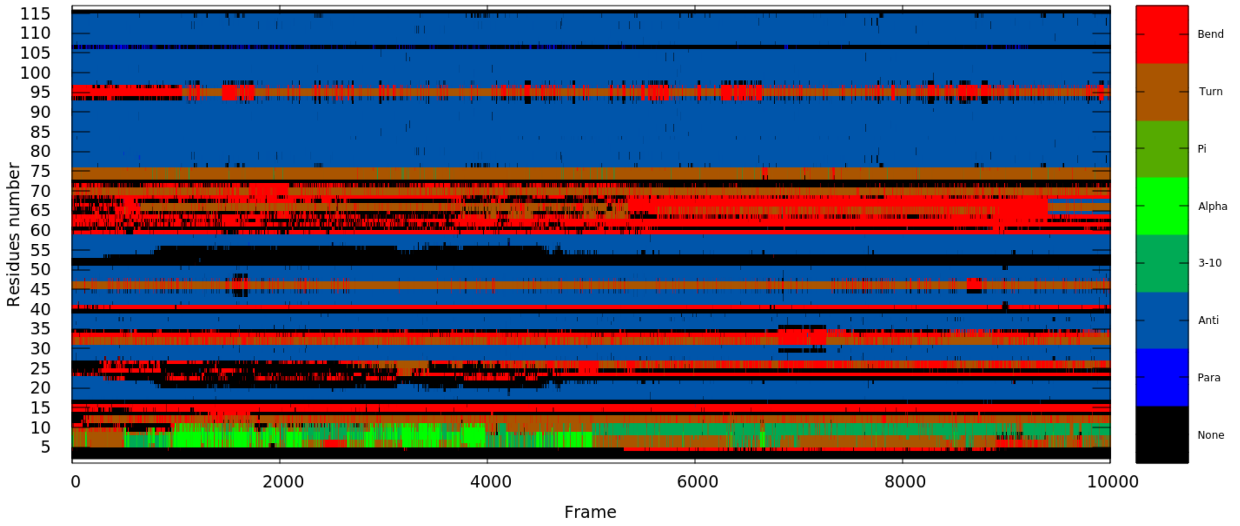

Figure S4. Per-residue energy decomposition analysis performed by averaging the results gained from each MD simulation executed on complex-S.

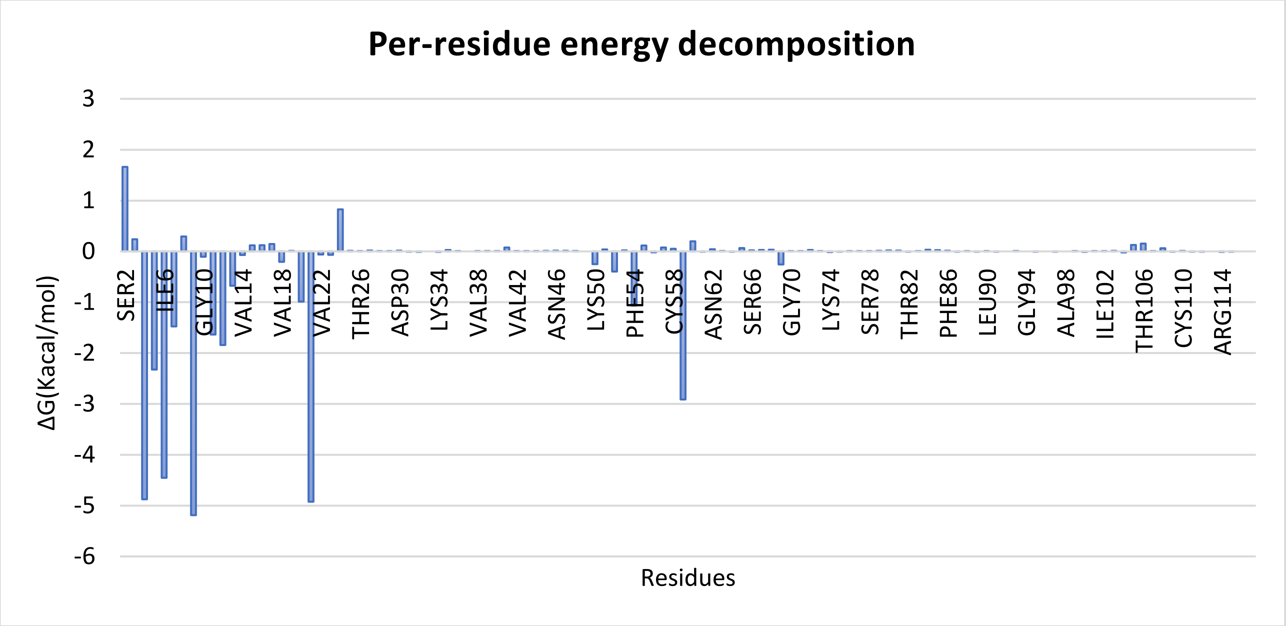

Table S2. Distances profiles of the key interactions detected during the three MD runs performed on complex-S. The selected distances were computed for all the frames and the results were averaged. The mean values over the three simulations were computed. All the distances are expressed in Å.

**Residue NGF**	**Residue TrkA**	**Complex-S MD1**	**Complex-S MD2**	**Complex-S MD3**	**Average**
H4@HE2	F303@O	2.07±0.34	2.05±0.30	2.22±0.52	2.11±0.39
H4@HE2	G344@O	3.34±0.74	3.15±0.62	2.96±0.83	3.15±0.73
H4@HE2	S304@OG	3.82±0.93	4.29±1.11	4.58±1.30	4.23±1.11
H4@HE2	H343@ND1	3.77±0.88	3.82±0.73	4.15±0.94	3.91±0.85
H4@imidazole	H291@imidazole	5.30±0.67	5.59±0.77	5.91±1.19	5.60±0.87
H4@imidazole	P302@pyrrolidine	5.02±0.28	4.98±0.30	5.00±0.29	5.00±0.29
P5@pyrrolidine	L333@CG	6.28±0.48	6.02±0.55	5.97±0.45	6.09±0.49
I6@CB	C300@SG	5.39±0.49	5.06±0.56	5.04±0.58	5.16±0.54
I6@CB	C345@SG	5.28±0.79	5.03±0.88	4.92±0.61	5.08±0.76
F7@phenyl	V294@CB	5.69±0.90	5.74±1.12	6.29±1.74	5.91±1.25
R9@CZ	E334@CD	4.14±0.61	6.88±3.99	5.26±2.10	5.43±2.23
E11@O	H297@H	3.85±2.04	4.29±1.40	3.77±1.90	3.97±1.78
E11@CD	R347@CZ	9.19±3.43	4.22±0.82	4.99±1.81	6.13±2.02
W21@HE1	H253@ND1	3.08±1.64	2.27±0.50	3.90±1.96	3.08±1.37
W21@HE1	D380@O	3.52±1.00	3.88±0.76	3.64±1.44	3.68±1.07
R59@CZ	E295@CD	5.73±2.03	10.03±2.87	4.88±1.34	6.88±2.08

Figure S5. Contribution of each energy term to the binding free energy of complex-S, complex-C and TrkA-NGF(1-14) obtained by averaging the results gained from each MD runs performed on the three complexes.
(VDWAALS = van der Waals energy, EEL = electrostatic energy, EGB= polar solvation energy, ESURF= non- polar contribution to the solvation free energy)

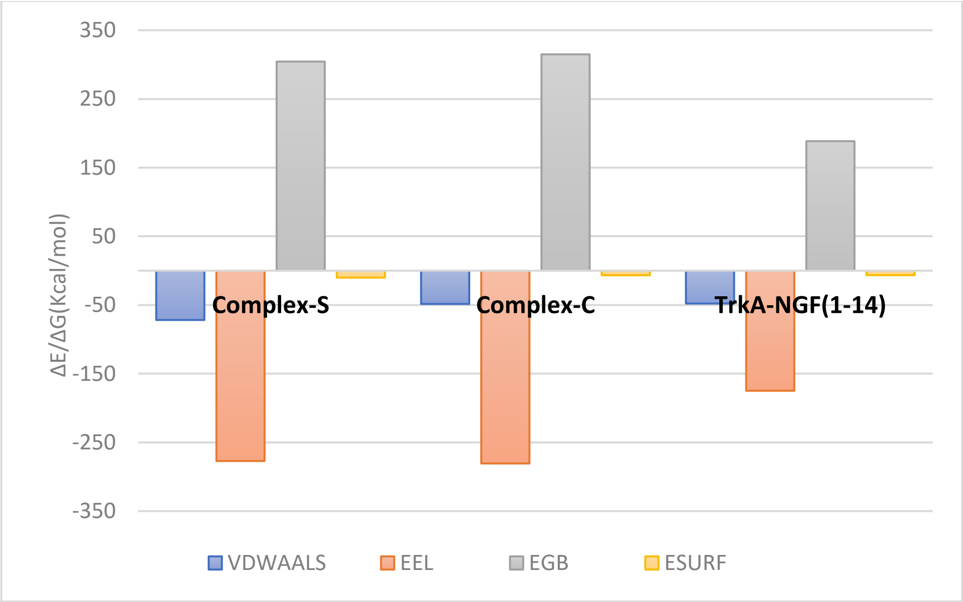

Figure S6. Per-residue energy decomposition results performed on TrkA-NGF(1-14) complex by averaging the outcomes obtained from each MD run.

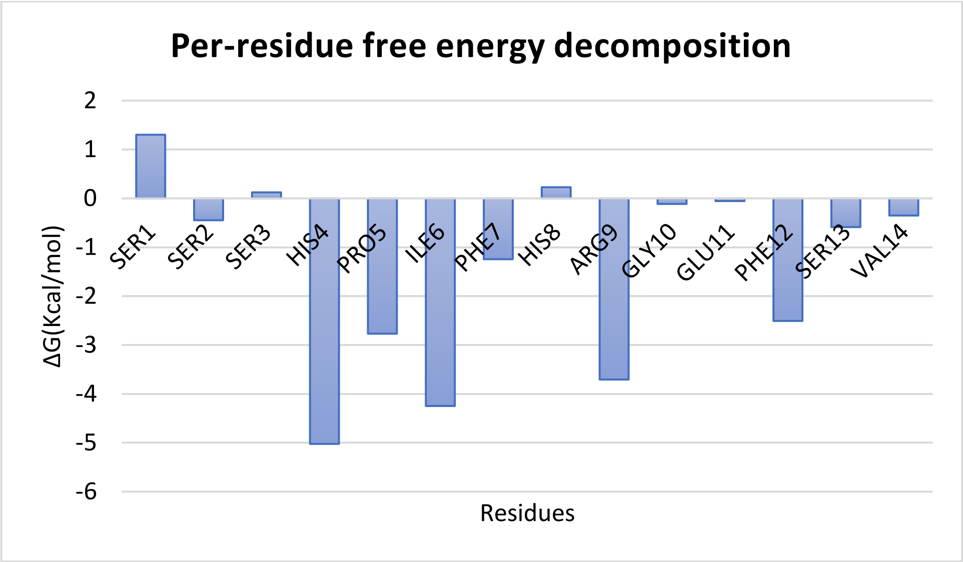

Table S3. Distances profiles of the key interactions detected during the three MD runs performed on NGF(1-14)-TrkA complex. The selected distances were computed for all the frames and the results were averaged. The mean value over the three simulation was computed. All the distances are expressed in Å.

Residue NGF(1-14)	Residue TrkA	NGF(1-14)-TrkA MD1	NGF(1-14)-TrkA MD2	NGF(1-14)-TrkA MD3	Average
H4@HE2	F303@O	2.29±0.51	2.65±0.49	2.08±0.36	2.34±0.45
H4@HE2	G344@O	2.79±0.76	2.17±0.46	3.59±0.78	2.85±0.67
H4@HE2	S304@OG	4.11±0.90	5.05±0.77	3.30±0.66	4.15±0.78
H4@HE2	H343@ND1	4.12±0.77	4.77±0.57	3.66±0.58	4.18±0.64
H4@imidazole	H291@imidazole	5.71±0.86	5.58±0.49	5.68±1.01	5.66±0,79
H4@imidazole	P302@pyrrolidine	4.93±0.24	4.96±0.26	4.90±0.22	4.93±0.24
P5@ring	L333@CG	5.86±0.42	5.72±0.44	6.13±0.70	5.90±0.52
I6@CB	C300@SG	5.26±0.74	5.47±0.59	5.14±0.47	5.29±0.60
I6@CB	C345@SG	5.15±0.74	5.64±0.69	5.11±0.56	5.30±0.66
F7@phenyl	V294@CB	5.96±0.97	8.80±2.00	5.68±0.51	6.81±1.16
R9@CZ	E334@CD	7.21±2.86	8.79±4.24	8.01±1.81	8.00±2.97
E11@O	H297@H	9.66±2.39	10.31±2.68	8.76±1.87	9.58±2.31
E11@CD	R347@CZ	10.70±3.65	15.89±3.22	5.51±2.67	10.70±3.18

Figure S7. Secondary structural analysis for NGF(1-14) in complex with TrkA. A) Average structural propensity over all frames for each residue. B) Time evolution of the secondary structure for each residue. C) Representative structure of NGF(1-14) in the bound state.

A

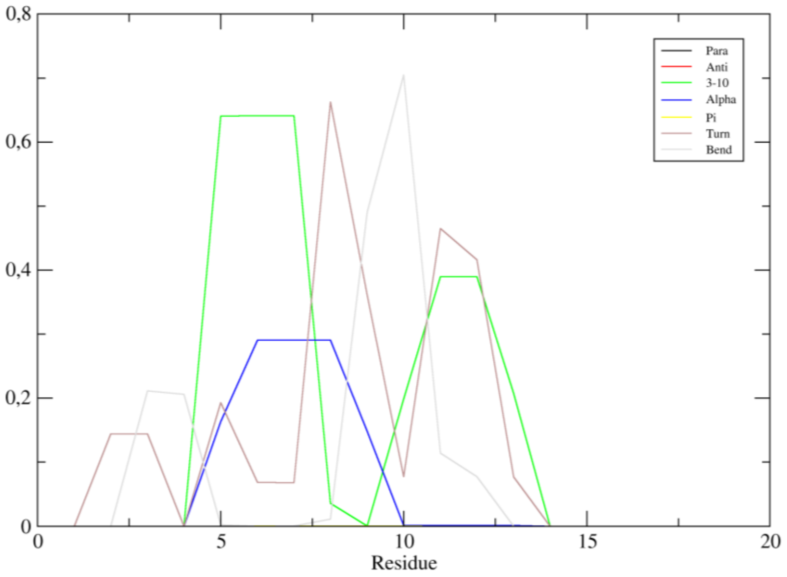

B

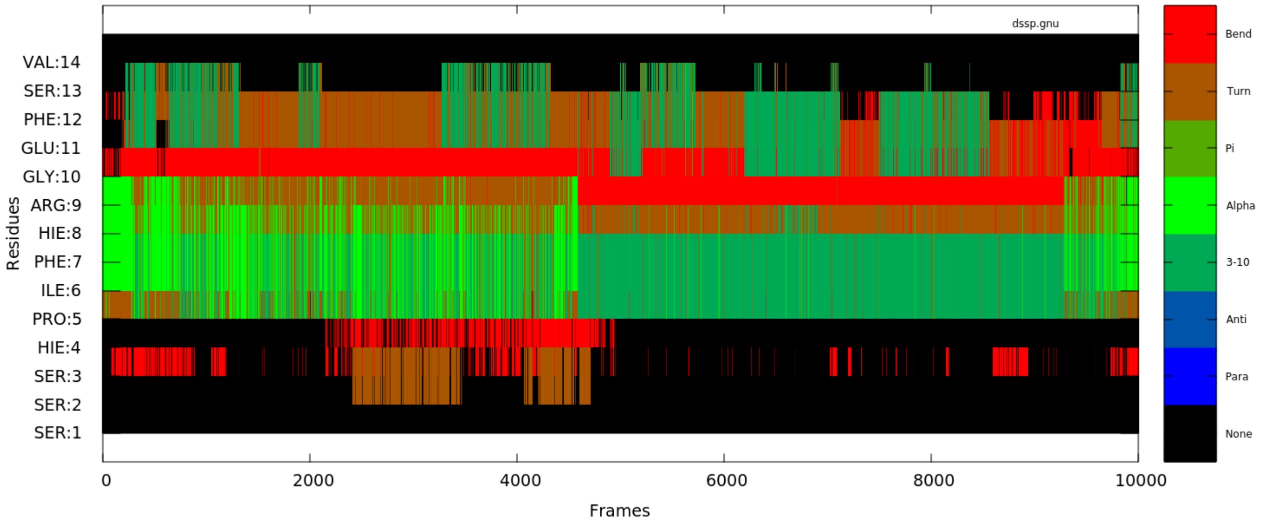

C

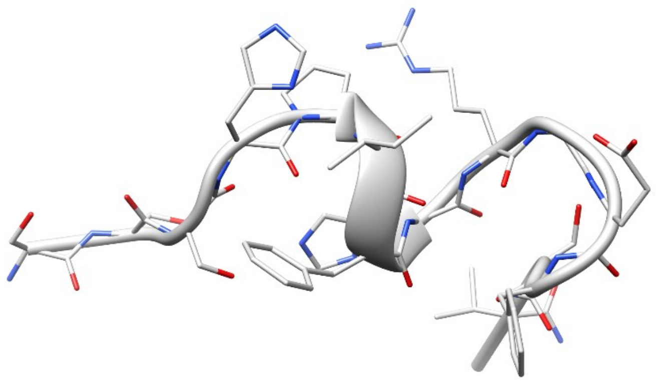

Figure S8. Secondary structural analysis for NGF(1-14) in aqueous solution. A) Average structural propensity over all frames for each residue. B) Time evolution of the secondary structure for each residue. C) Representative structure of NGF(1-14) in solution.

A

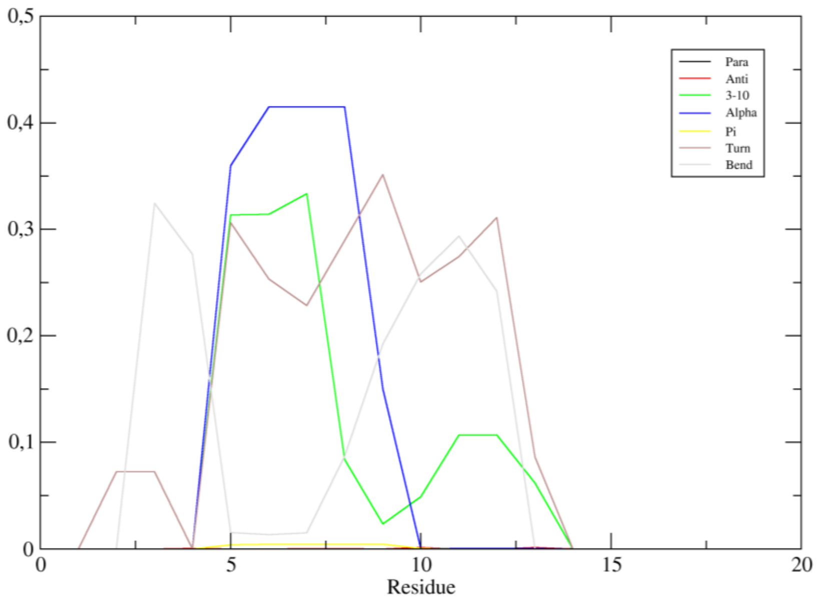

B

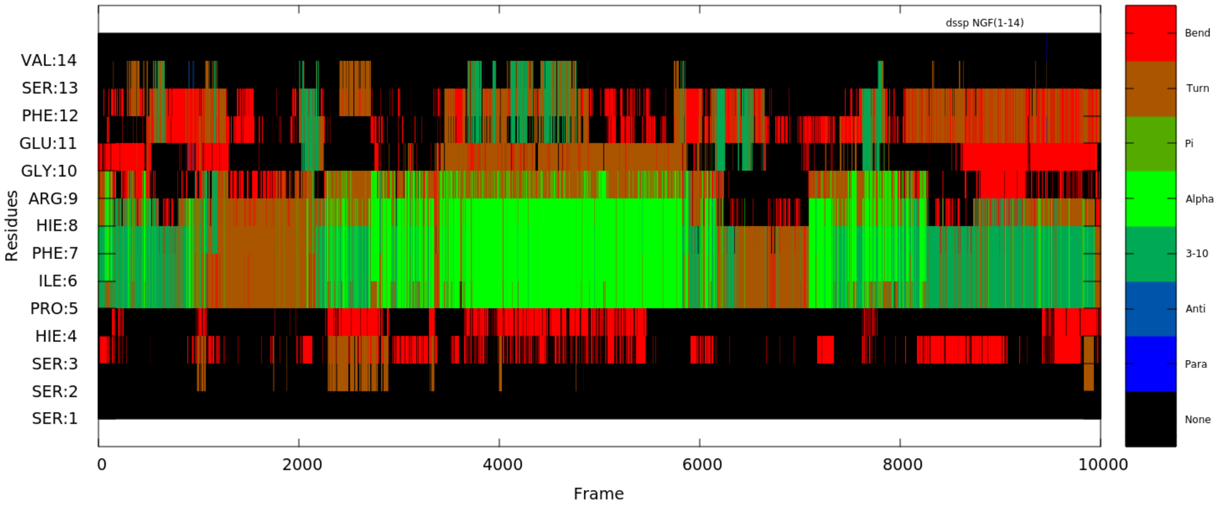

C

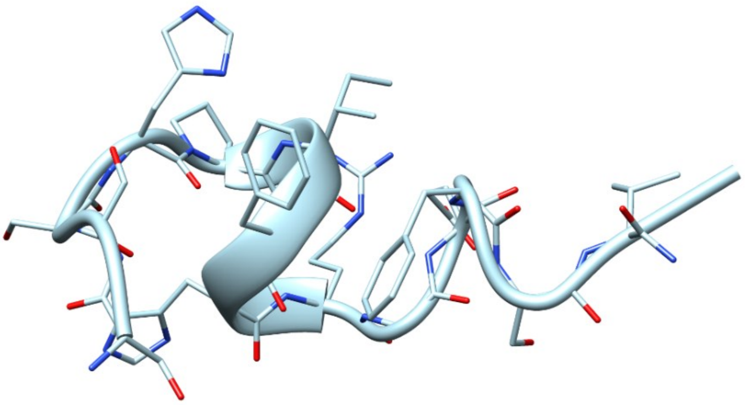

Figure S9. RMSF profiles of TrkA in complex with the mutated peptides.

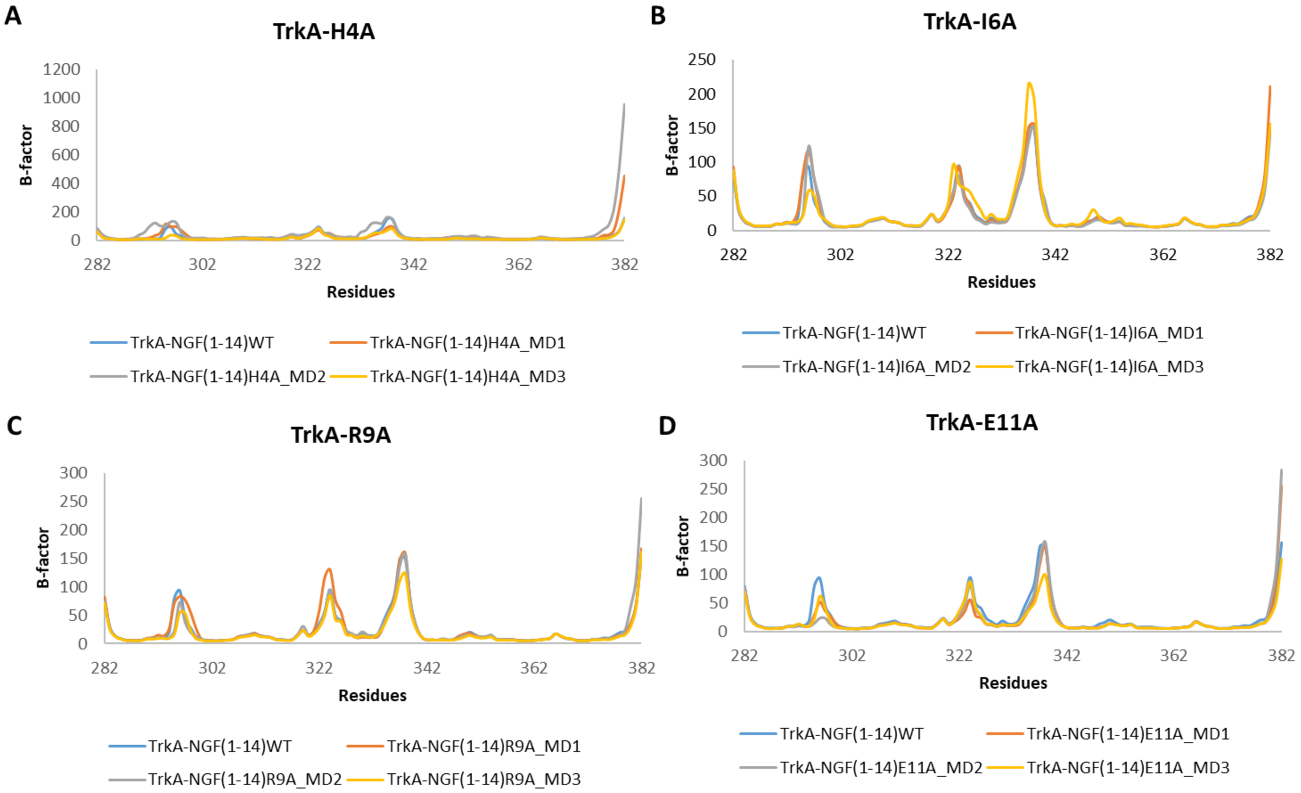

Figure S10. Contribution of each energy term to the binding free energy of the complexes involving the mutated peptides. (VDWAALS= van der Waals energy; EEL = electrostatic energy; EGB = polar solvation energy; ESURF= non-polar contribution to the solvation free energy).

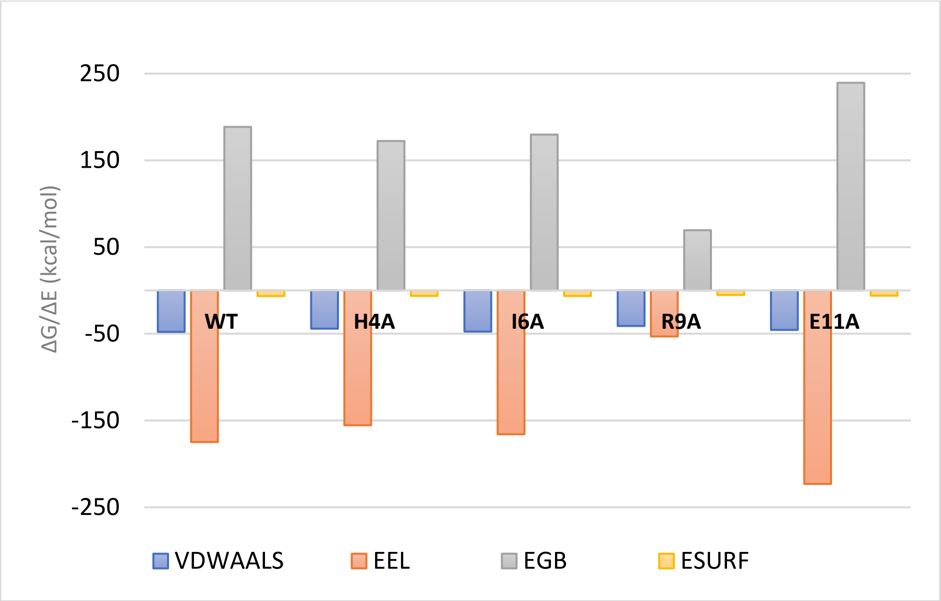

Supplement: Supplementary file 1 [file cells-11-02808-s001.zip › cells-1867837-supplementary.pdf]
